# Integrating Optical Genome Mapping and Whole Genome Sequencing in Somatic Structural Variant Detection

**DOI:** 10.3390/jpm14030291

**Published:** 2024-03-09

**Authors:** Laura Budurlean, Diwakar Bastihalli Tukaramrao, Lijun Zhang, Sinisa Dovat, James Broach

**Affiliations:** 1 Department of Biochemistry & Molecular Biology, Penn State College of Medicine, Hershey, PA 17033, USA; 2 Department of Pediatrics, Penn State Cancer Institute, Hershey, PA 17033, USA; 3 Department of Population & Quantitative Health Sciences, Case Western Reserve University, Cleveland, OH 44106, USA

**Keywords:** Bionano, optical genome mapping, whole genome sequencing, next generation sequencing, RNA sequencing, B-ALL, leukemia, structural variation

## Abstract

Structural variants drive tumorigenesis by disrupting normal gene function through insertions, inversions, translocations, and copy number changes, including deletions and duplications. Detecting structural variants is crucial for revealing their roles in tumor development, clinical outcomes, and personalized therapy. Presently, most studies rely on short-read data from next-generation sequencing that aligns back to a reference genome to determine if and, if so, where a structural variant occurs. However, structural variant discovery by short-read sequencing is challenging, primarily because of the difficulty in mapping regions of repetitive sequences. Optical genome mapping (OGM) is a recent technology used for imaging and assembling long DNA strands to detect structural variations. To capture the structural variant landscape more thoroughly in the human genome, we developed an integrated pipeline that combines Bionano OGM and Illumina whole-genome sequencing and applied it to samples from 29 pediatric B-ALL patients. The addition of OGM allowed us to identify 511 deletions, 506 insertions, 93 duplications/gains, and 145 translocations that were otherwise missed in the short-read data. Moreover, we identified several novel gene fusions, the expression of which was confirmed by RNA sequencing. Our results highlight the benefit of integrating OGM and short-read detection methods to obtain a comprehensive analysis of genetic variation that can aid in clinical diagnosis, provide new therapeutic targets, and improve personalized medicine in cancers driven by structural variation.

## 1. Introduction

Structural variants (SVs) are alterations that affect large segments of genomic DNA (>50 bp) and can act as genetic drivers in many cancers [1]. Since deletions, duplications, inversions, translocations, insertions, and copy number variants (CNVs) can influence gene expression or function, they often serve as prognostic indicators in human cancers [2,3,4,5]. A primary challenge with traditional SV detection methods is large repetitive genomic sequences, which comprise two thirds of the human genome and confound sequencing using short-reads [6,7]. We report here that optical genome mapping (OGM) can complement whole-genome sequencing (WGS) in the detection of SVs, and their combination provides an enhanced landscape of SVs compared to either alone [8].

OGM interrogates ultra-high molecular weight DNA that is extracted from cells or tissues and labeled with a DL-Green fluorophore covalently attached to the DNA at specific consensus sites [9,10]. Labeling creates a unique barcode of the genome while preserving the integrity of genomic DNA. After labeling, the DNA is linearized by extrusion into nanochannel arrays where each labeled molecule is imaged and converted into optical maps that can be assembled into local contiguous segments or a complete genome de novo and then compared to a reference genome to detect any structural abnormalities. The ultra-long reads allow direct visualization of SVs rather than relying on inferred identification by short-read sequencing [11,12]. 

Several long-read WGS methods are capable of detecting SVs, such as Oxford Nanopore (ONT), 10X Genomics linked-reads, or Pacific Biosciences (PacBio) HiFi, which yield SV detection at a level comparable to that of OGM. The main advantage of using long-read sequencing is to obtain simultaneous detection of both SVs and single nucleotide variants (SNVs). However, ONT and PacBio suffer from an inferior base calling accuracy in sequencing, making them less suitable for SNV applications compared to short-read sequencing methods such as Illumina [13].

As an alternative to long-read sequencing as a means of capturing both SVs and SNVs, we have explored the synergy between short-read WGS and OGM not only for identifying both SVs and SNVs but also for enhancing SV detection. The studies comparing OGM to current long-read next-generation sequencing (NGS) technologies highlighted the differences between these two orthogonal methods [9,12,14,15]. Largely left out of the discussion was short-read NGS data and how they might integrate with OGM. The few existing studies to research this only examine single individuals [16,17]. To date, most studies have mainly been carried out with a combination of WGS and cytogenetics but have failed to address the SVs invisible to both WGS and cytogenetics that may be detected by OGM. For example, G-banding has been used to detect SVs in bone tumors. The major drawback of this method is low resolution with most events requiring >5–10 Mb in size to be detected. Aneuploidy is reported as a result of the Bionano optical genome mapping rare variant pipeline based on coverage over a certain region with events as small as 380 kb being detectable. A 5% allele frequency aneuploidy is detectable in most chromosomes. At a 9% allele frequency, all but chrY have a sensitivity of >95% [18]. Previously, Dixon et al. showed that a combination of high-throughput chromosome conformation capture (Hi-C), WGS, and OGM identified SVs that were undetectable by typical cytogenetic tools, such as karyotyping, for cancer genome analysis in several cell lines [19]. A follow-up study from the same group looked at SVs in 12 leukemia patients using a combination of WGS and OGM and obtained similar results [20]. Here, we examine 29 pediatric B-ALL patients with short-read WGS and OGM and introduce a computational pipeline for integrating the two techniques for somatic SV detection in the absence of control DNA. We determined that only 11.6% of SVs are detected by both methods and we examine the areas and reasons of nonconcurrence. These results suggest that integrating OGM with WGS provides high sensitivity for SV detection and may aid detection in clinical diagnostics.

## 2. Materials and Methods

- Patient population

The samples were obtained from patients diagnosed with B-ALL and younger than 20 years old at the time of diagnosis (Supplementary Table S1). The individual samples were sourced from collaborators at various institutions including Loma Linda University (Loma Linda, CA, USA), University of Southern California Keck School of Medicine (Los Angeles, CA, USA), St. Jude’s (Los Angeles, CA, USA), Childrens Cancer Institute (Sydney, Australia), and from Dr. Markus Müschen, City of Hope Beckman Research Institute (Duarte, CA, USA) in compliance with institutional review board regulations.

- Development of PDX models

For the development of the primary human B-ALL mouse xenograft model, 2.5 × 10^6^ patient B-ALL cells per mouse were transplanted intravenously into 5–8-week-old female NOD.Cg-PrkdcSCID Il2rgtm1Wjl/SzJ (NSG) mice, as previously described [21]. All of the animal experiments were conducted in the Developmental Therapeutics Preclinical Core facility at Penn State University College of Medicine under protocols approved by the Institutional Animal Care and Use Committee at Penn State University College of Medicine (Hershey, PA). Aliquots of single cell suspensions from the bone marrow or spleen of each mouse were blocked with human CD16/32 antibodies (Biolegend) and then stained for flow cytometry with antibodies specific to mouse CD45-FITC (#103108; Biolegend), human CD45-APC-Cy7 (#368516; Biolegend), human CD19-APC (#302212; Biolegend), human CD33-BUV661 (#750435; BD Biosciences), human CD10-PE (#312204; Biolegend), and human CD3-BV421, (# 562426; BD Biosciences). The resulting cells were analyzed by flow cytometry (BD FACSymphony A3 Cell Analyzer), as previously described [21]. The CD45 cell engraftment in animal bone marrow (BM) and spleen (SP) data are shown in Supplementary Figure S2.

- DNA isolation

The genomic DNA for OGM and WGS was extracted from bone marrow or spleen frozen cell pellets following the Bionano Genomics Prep SP Frozen Cell Pellet DNA Isolation protocol. Specifically, 1.5 × 10^6^ cells were used in optical mapping applications and 1 × 10^6^ were reserved for WGS libraries, and 0.5 × 10^6^ were reserved for RNA-Seq.

- Optical Mapping

Ultra-high molecular weight DNA (typically > 250 kb) was prepared for Bionano optical genome mapping on the Saphyr instrument, as previously described [22]. SV analysis was performed with the rare variant pipeline (RVP) in Bionano Access v1.6 and Bionano Solve 3.6 using the default parameters. DLE-1 was used for direct labeling and staining for OGM applications following the Bionano Prep Direct Label and Stain (DLS) protocol. We imaged to an average of 273X coverage (min: 228, max: 344) with an average label density of 15.2/100 kb (min: 14.2, max: 25.4). The average mapping rate was 72.0% (min: 51.7, max: 90.6).

- Whole Genome Sequencing

The DNA from PBMC was purified using DNeasy Blood/Tissue Kit (Qiagen) in accordance with the manufacturer protocol. The gDNA was sheared to 400 bp using a Covaris sonicator if required by the library preparation method. The WGS libraries were prepared using either KAPA HyperPrep PCR-free Kit (Roche) or the DNA PCR-Free Prep, Tagmentation kit (Illumina) according to the manufacturer instructions. The samples were sequenced using Illumina Novaseq S2 or S4 150 bp paired-end sequencing at 40X coverage.

- RNA-Seq

The total RNA was extracted using the Novogene (Davis, CA, USA) recommended workflow with TRIzol reagent. Downstream sample preparation and RNA-Seq were performed by Novogene (Davis, CA, USA). We obtained a total of ≈53.3 million RNA-Seq reads per sample replicate (*n* = 3) and retained fusion events in the analysis if they had ≥16 supporting hybrid reads, unless otherwise noted.

- SV workflow for WGS

Alignment to hg38 was performed using SpeedSeq (version 0.1.2). We utilized the built-in SV caller LUMPY and the SNV caller Freebayes within the SpeedSeq pipeline. We also separately called SVs utilizing DELLY (version 0.8.1). The CNVs were called using FREEC (version 11.5) using a window size of 50 kb [23,24].

In the absence of normal control gDNA, we employed a filtration pipeline to reduce false positive calls from DELLY and LUMPY as follows: first, all SV calls had to be supported by at least three split reads (SR) or three spanning paired-end reads (PE). We required at least five supporting reads to call a translocation. We marked WGS gene fusions as putative if they overlapped a repeat region and were not supported by a second method, either OGM or RNA-Seq. SVs <50 bp were removed, in addition to SVs that mapped to chromosome Y or to the mitochondrial genome. SV calls from DELLY and LUMPY were merged, and SVs that were identified by both methods were retained. We used separate criteria to call SVs overlapping between the two methods depending on the type of SV. For deletions, the calls were merged between the two pipelines if they had reciprocal overlap (RO) ≥50%. We used the coordinates provided by LUMPY for this merged deletion set. For inversions, the calls identified by both LUMPY and DELLY were merged if they had an RO ≥0.9. The final merged coordinates were based on the coordinates from the LUMPY calls. Translocations were merged between the two pipelines if the paired break ends mapped within +/− 50 bp of each other and if the strand of the break ends matched. The final coordinates were based on the calls from LUMPY. Regions annotated as insertions were identified by DELLY; LUMPY does not detect insertions. The deletions were removed if they had at least 50% reciprocal overlap (RO ≥ 50%) with known gap regions within 50 bp or overlap a centromere region.

- Integrating WGS and OGM variants

We integrated SVs from WGS and OGM as follows. We first defined strand orientation for SVs detected from different methods: “+” indicates the breakpoint located at the 3′ end of the joined arm, and “−” indicates the breakpoint at the 5′ end of the joined arm. In WGS, this dictates that SVs originally classified as deletions are given the strand orientation of “+−”, inversions as “++, and −−”, duplications as “−+” and unclassified intra-chromosomal rearrangement as “++” or −−”. Optical mapping originally reports deletions, which are assigned a strand orientation of “+−”, and inversions, which are assigned as “++” or “−−”. To determine whether the SVs detected by different methods reflect the same event, we set conditions when comparing inter-chromosomal translocations with large intra-chromosomal SVs: (1) They had the same location at both ends of the break point. (2) They had the same strand direction. Since the different methods have different resolutions for SV detection, we use variable criteria to determine whether two methods identify SVs in the “same locations”. This overlap is set so that breakpoints within +/− 500 kb are considered overlaps when comparing WGS and OGM. We determined deletions as overlapping if at least 50% of the deletion defined by WGS overlaps with the deletion defined by OGM, and the size of the deletion from OGM data must be 80–120% of the total length detected by WGS. In comparing smaller scale SVs, we found that insertions detected by optical mapping are sometimes resolved as duplications in WGS, which we annotate as duplications. Similarly, some WGS translocation calls are annotated as insertion calls in OGM data when one breakpoint cannot be resolved, such is the case in gap regions, centromeric, telomeric, or certain repetitive regions. More stringently, we considered an OGM insertion call to map to a WGS translocation call if the OGM insertion was within 100 kb of the WGS breakpoint. We reported SV breakpoint coordinates by the higher resolution method, WGS. Due to the nature of leukemia samples, a normal control, typically generated using a patient’s blood, is not available to filter out germline SVs. Somatic SV calls were thus made by excluding SVs found in Bionano’s control database and the Database of Genomic Variants (DGV) to filter out known genomic polymorphisms [25].

- Annotation of SV files

The annotation of BED files with SV position information was carried out using AnnotSV to provide functional, regulatory, and clinically relevant information about SVs [26]. SV waterfall plots were generated with D3Oncoprint version (version 1.0). The annotation of translocation SV files was carried out using a naïve Bayes classifier, Oncofuse (version 1.1.1), to predict the oncogenic potential of fusion genes and annotate breakpoints [27]. The in-house Bionano rare variant annotation pipeline was used to annotate the putative gene fusion events found by OGM.

- Statistics

The associations among the categorical values were examined using the Chi-square test or a two-sided Fisher’s exact test. Testing for differentially mutated genes was carried out by using Fisher’s exact test with a Benjamini Hochberg correction when applicable for false discovery rate. Nonparametric size distributions of SVs were analyzed using the Wilcoxon rank-sum test. Analyses and chart production were performed by using R version 4.1.3, GraphPad Prism version 9.5.1, Excel version 2302, or GPower version 3.1, *p* < 0.05 was considered statistically significant unless otherwise noted.

## 3. Results

### 3.1. Identification of Somatic Structural Variants in B-ALL

We used a combination of whole genome sequencing and optical mapping to identify structural variants in xenografts from 29 pediatric patients 20 years of age or younger with B-cell acute lymphoblastic leukemia (B-ALL) (Supplementary Table S1). We performed whole genome sequencing on all samples at an average depth of 40X and optical mapping at an average coverage of 273X (220X − 344X) on a Bionano Genomics Saphyr optical mapping instrument. For optical mapping, large genomic fragments (>250 kb) were extracted from cells, fluorescently labeled with a site-specific DNA binding protein, and then electrophoresed through nanochannels of a Saphyr chip that forces the molecules into a strictly linear conformation. As they migrated through the nanochannels, the DNA molecules were imaged, with the fluorescent tags providing a bar code that allowed subsequent assembly of individual molecules into larger contiguous maps, which were compared to a reference genome to identify insertions, deletions, and rearrangements. Finally, we performed RNA sequencing on all samples as a means of confirming a subset of SVs identified from OGM and WGS, particularly those that were predicted to generate gene fusions. As noted in the following, the combination of the three methods provided a significantly more robust means of retrieving SVs than any single method alone.

**Figure 1:**
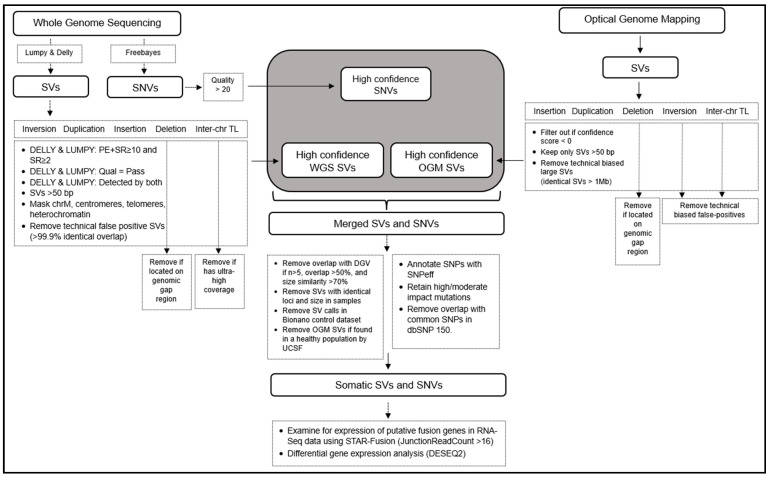
Bioinformatics workflow for structural variant detection and integration of whole genome sequencing, optical genome mapping, and RNA-Seq: SVs from both WGS, OGM, and RNA-Seq are detected and filtered using the individual subroutines depicted in the dotted boxes. High-confidence calls are merged together, and further filtering is applied to remove germline polymorphisms and SV calls from control datasets. The remaining calls represent likely somatic SVs and SNVs. Note that in the absence of germline controls for these individuals, true somatic calls cannot be fully determined, and we are limited by the comprehensiveness of the control datasets that are available from healthy control populations. A detailed explanation is provided in Material and Methods.

Our bioinformatics workflow identified SVs in WGS data through application of the LUMPY and DELLY software packages and SVs in OGM data using the Bionano Access rare variant pipeline (RVP), as illustrated in (Figure 1). We retained SVs from WGS only if they were identified by both LUMPY and DELLY, with the exception of insertions since LUMPY does not detect insertions. Since we did not have access to germline DNA from the leukemia patients, we filtered SVs and SNVs in order to eliminate most germline SVs by comparing them to the Database of Genomic Variants (DGV). SVs matching >50% in overlap and >70% similar in size in ≥5 individuals in DGV were considered polymorphisms and were removed. SVs in the Bionano control dataset were similarly removed, as well as any SVs present in a healthy control population as curated by the University of California San Francisco [28]. Finally, the presence and expression of any gene fusions were verified with RNA-Seq. A combination of three technologies provided robust identification of SVs due to the different capabilities of the different technologies for detecting SVs (Supplementary Table S2). A detailed explanation of the bioinformatics workflow is provided in the methods section.

### 3.2. Discordance of WGS and OGM in Somatic SV Detection

**Figure 2:**
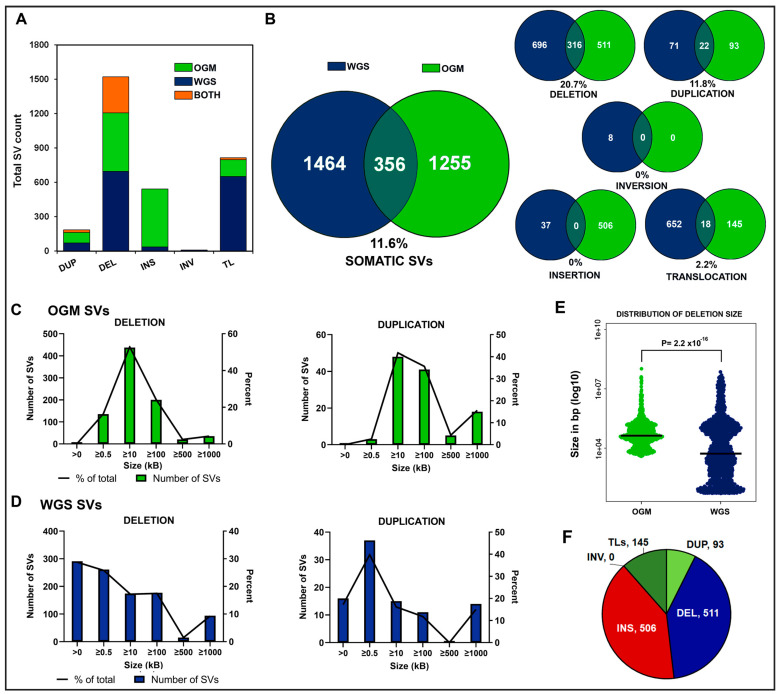
The SV landscape in B-ALL as detected by WGS and OGM technologies in 29 individuals with pediatric B-ALL. (**A**) Average number of somatic SVs detected by each technology per sample and the average overlap per sample genome. (**B**) Counts of somatic SVs detected in cohort by WGS or OGM and percent confirmed by both technologies grouped by SV type. (**C**) Distribution of counts and percent of somatic deletions and duplication regions detected at various sizes by OGM from <0.5 kbp to ≥1 Mb. No inversions were detected by OGM in our 29 individuals. (**D**) Distribution of counts and percent of somatic deletions and duplication regions detected at various sizes by WGS from <0.5 kbp to ≥1 Mb. Insertion sizes for WGS are not reported by DELLY/LUMPY and are not included here. (**E**) Distribution of deletion sizes. The median sizes shown as horizontal lines are 42,720 for OGM and 5248 for WGS. The values are plotted on a log10 scale. *p* = 2.2 × 10^−16^ Wilcoxon rank-sum test. (**F**) The number of SVs found in 29 pediatric B-ALL cases by OGM that were not detected by WGS. TLs = translocations, DEL = deletion, INS = insertions, DUP = duplication, INV = inversions.

We compared the types and quantities of somatic SVs detected by short-read WGS and OGM technologies individually and the overlap of the two in our 29 samples (Figure 2A). We observed an average of 11.6% overlap between SVs detected by the two technologies across these individuals. This included 20.7% overlap in deletions, 11.8% in duplications, 2.2% in translocations, and 0% overlap in inversions or insertions (Figure 2B).

This striking discrepancy in SV identification by the two methods likely has a variety of sources. First, an SV could be misclassified. For instance, a translocation called by WGS could be a relatively short segment of one chromosome inserted into another chromosome. If that inserted segment is less than 50 kb or so, OGM would not be able to identify its origin and would label it an insertion, while WGS would identify it as one (or two) translocation(s). Second, one of the methods could be in error. For instance, WGS might call a translocation at the site of an insertion element, due to the repetition of the insertion element at other sites in the genome, even though no such translocation was present. OGM would not identify that region as a translocation. Third, one of the methods might not be able to detect an event identified by the other method. For instance, regions of low DSE-1 label density, such as paracentric or subtelomeric regions, are not well mapped by OGM and SVs located or initiated in those regions would be missed by OGM. Similarly, LUMPY and DELLY are admittedly poor at identifying insertions and report only a very limited number. These sources of discrepancy between the two methods are discussed more fully in the next section.

Besides the sources of discrepancies in SV identification between the two methods suggested above, we found that the methods have different sensitivities for detecting SVs of different sizes (Figure 2C–E). In general, OGM identified more larger (>10 kb) deletions and duplications than WGS, while WGS identified more smaller deletions and duplication than OGM. We did not compare insertions by size in this study due to the limitations of short-read SV callers such as LUMPY and DELLY in identifying insertions and reporting their sizes. We also did not compare inversion sizes since no inversions were reported in the OGM data after filtering, and only 9 inversions were detected in the entire cohort by WGS (Figure 2C,D). We note that neither OGM nor WGS can differentiate a translocation between homologous chromosomes from an inversion occurring at the same breakpoints, and there may be instances when intra-chromosomal translocation calls are actually inversions and vice-versa. Finally, acknowledging that WGS is most often the method of choice for genomic or somatic SV identification, we found that OGM detected an additional 511 deletions, 506 insertions, 93 duplication/gains, and 145 translocations across our 29 pediatric B-ALL individuals, which highlights the benefit of incorporating OGM data into SV detection (Figure 2F).

Since our studies were performed on PDX cells propagated in mice, we compared SVs in three PDX samples for which we had access to the primary patient tumor cells (Supplementary Figure S1). PDX models are an established tool for amplifying those subpopulations in patient tumors carrying driver mutations, particularly those present at a low variant allele fraction in heterogenous tumors that might be missed in lower coverage situations. Furthermore, PDX models can help prioritize SVs for targeted therapies and generally accurately recapitulate the mutations present in a patient tumor [29]. Across our three samples, we found that an average of 5 out of 53 SVs were lost in the PDX model, while 5.3 SVs were gained. Those that were lost were on average at a lower allele frequency (VAF = 0.33) than those acquired in the PDX model (VAF = 0.47) (Supplementary Figure S1C). The SVs retained in both primary and PDX models have similar allele frequencies (*p* = 0.31) with the highest density at around 0.5 VAF and a slight skew toward higher VAFs in the PDX model. (Supplementary Figure S1D). We present CD45 cell engraftment in animal bone marrow (BM) and spleens (SP) in Supplementary Figure S2. In summary, we see that the PDX model predominantly recapitulates the tumor structural variant landscape and reveals some SVs not readily evident in the primary tumors.

### 3.3. Resolution of Unsupported Breakpoints

**Figure 3:**
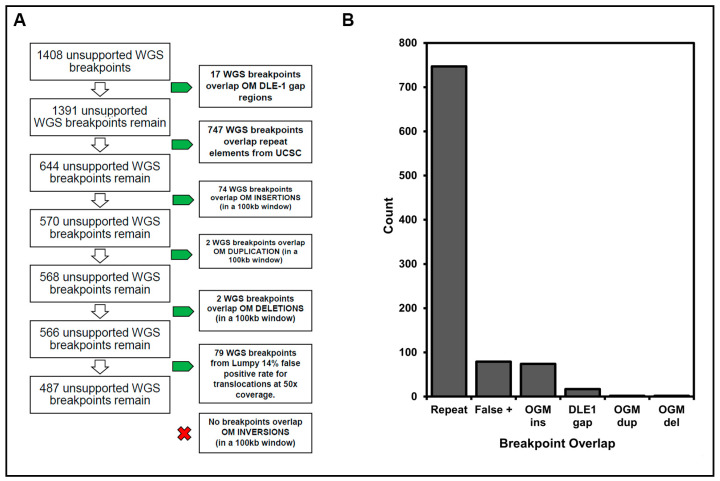
Resolution of unsupported WGS breakpoints by OGM data: (**A**) We examined likely sources for breakpoint discrepancies in SVs detected by OGM and WGS, including DLE-1 gap regions, UCSC known repeat regions, WGS SVs overlapping OGM SVs within 100 kb, and any predicted false positive translocation calls from LUMPY. (**B**) The number of unresolved WGS breakpoints that overlap with UCSC repeats, OGM insertions (ins), duplications (dup), and deletion (del) regions, DLE-1 gap sites, or otherwise likely false positive translocation calls from LUMPY.

To gain a better understanding of the sources of the discrepancies between OGM and WGS, we examined in detail the 1408 breakpoints reported by WGS that were unsupported by a corresponding breakpoint in OGM data. We applied a multi-step filtering process to identify sources of discrepancy as outlined in Figure 3. First, several genomic locations are known to have sparse DLE-1 labeling sites. Accordingly, OGM would not identify SVs in those regions. This likely accounts for 17 breakpoints at which WGS made a call that OGM failed to confirm. Second, we observed that 747 (53%) of the unsupported WGS breakpoints overlapped with a repeat element from the UCSC data. Resolving SVs over repeat regions is especially challenging to accomplish using short-read WGS [6,7,11,22,23]. Accordingly, WGS analysis could misinterpret sequence reads with a single LINE, SINE or ALU element as a translocation between two identical repeat elements located at different genomic sites. Third, 74 WGS breakpoints overlapped OGM insertion calls, suggesting that the inserted sequences derived from a separate chromosome but was too short for OGM to identify its source. Similarly, two WGS breakpoints overlapped OGM duplications and another two overlapped OGM deletions. Finally, noting that LUMPY has a ~14% false positive rate for translocations at ~50X genome coverage and DELLY has a 3× higher false positive rate than LUMPY, we used LUMPY’s false-positive rate to further refine unresolved WGS breakpoints. This would potentially eliminate an additional 79 breakpoints from the remaining 566 unsupported WGS breakpoints, leaving 487 out of 1408 (34.6%) WGS breakpoints that are not supported by OGM data and 921/1408 (65.4%) that are likely explained (Figure 3).

**Figure 4:**
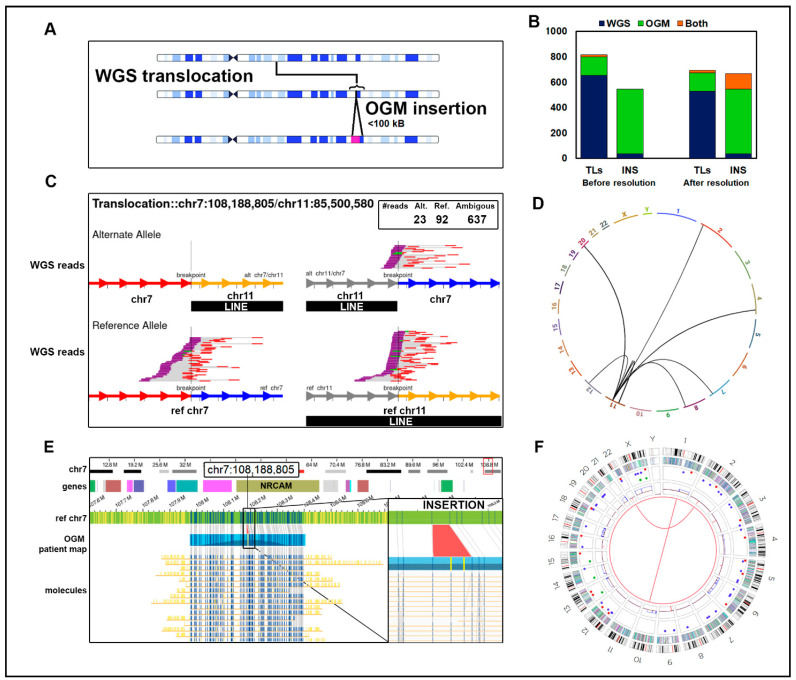
Insertions misclassified as translocations: (**A**) A translocation identified by WGS for which one end map near the site of an insertion called by OGM is likely misclassified, particularly if that insertion is a repeated element. (**B**) Counts of translocations and insertions in our cohort of 29 B-ALL samples before and after reclassifying as insertions 123 WGS translocations mapped to an OGM insertion region. (**C**) WGS data in sample ALL-02 of a false positive translocation between chr7 and chr11, which overlaps a LINE element on chr11. WGS reads indicating the translocation t(7;11) causing a putative NRCAM:DLG2 gene fusion. The box shows that 23 reads in the WGS data support a translocation (alternate allele) and 92 support the reference allele, but an additional 637 reads are ambiguous and do not support the reference or alternate allele calls. The reference allele is depicted on the bottom. Reads aligning to the minus strand are red, and those aligning to the plus strand are purple. The unsequenced segment between read pairs is depicted by gray bars. Green reads designate overlap of mate pair reads. Chr7 is depicted by red (upstream) and blue (downstream) arrows at the breakpoint. Chr11 is depicted by grey (upstream) and yellow (downstream) arrows at the breakpoint. (**D**) The circos plot derived from WGS data showed additional putative translocations from the LINE element in chr11 to other chromosomes. The t(7;11) is also depicted. (**E**) The OGM data from chr7 at the site of the predicted fusion breakpoint. The tracks depicted (top to bottom) are as follows: cytoband, hg38 genes, reference chromosome, patient map aligning to reference, and supporting long-read molecules. The vertical blue lines represent DLE-1 labels corresponding to the patient map. No evidence of a translocation is seen in the OGM data, but an insertion is shown in the zoomed-in panel highlighted in red. The two yellow vertical lines represent additional DLE-1 labels not originally present in the reference chr7. We hypothesize that the insertion is a LINE, which accounts for the alternative reads in the WGS data. (**F**) The OGM circos plot depicts which translocations pass filtering. The track labels are as follows from outside to inside: chromosome number, cytoband, gene regions, SV (blue = deletion, red = insertion, pink = inversion, green = duplication), copy number, and translocations.

Applying this analysis to translocations, we noted that WGS called a total of 652 translocations and OGM identified 145, but only 18 (2.2%) were resolved by both technologies. However, 123 of the 652 WGS translocations (19%) were mapped within 100 kb of an OGM insertion (Figure 4A). Of those, 45 (36%) WGS translocations had a breakpoint overlapping with a repeated element. An example of this situation, which we observed in several individuals, is outlined in Figure 4. A t(7;11) called by WGS corresponds to the site of an insertion, but no translocation, in a subset of the reads on this region of chr7 in OGM data (Figure 4E,F). The WGS translocation call is based on 23 reads linking the LINE element sequences on chr11 to unique sequences on chr7, compared with 92 reference calls over the corresponding regions on chr7 and chr11 (Figure 4C). Moreover, WGS returns 637 ambiguous calls for this region of chr11, which supports translocation calls from this site on chr11 to six other sites in the genome, all of which correspond to LINE insertions (Figure 4D). Our interpretation is that the 6.1 kb insertion on chr7 is a LINE, which WGS incorrectly maps to the chr11 LINE. After applying this analysis as a means of resolving the WGS translocations to nearby OGM insertions, we reassigned 123 SVs previously defined by WGS as translocations to insertions. This increased the concordance between the two methods (Figure 4B).

### 3.4. Comparison of OGM and WGS to Cytogenetic Data

**Figure 5:**
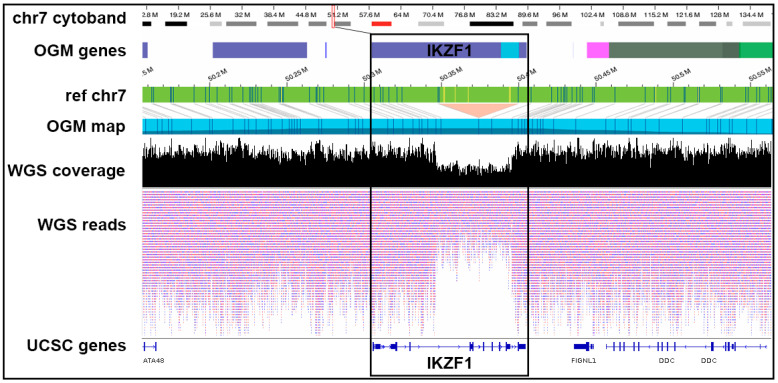
Smaller SVs are missed by conventional cytogenetics but can be accurately resolved by OGM and WGS: An example of a heterozygous partial IKZF1 deletion not reported by cytogenetics but confirmed by OGM and WGS data in LUMPY/DELLY is in a Hispanic patient, W31. The first four tracks depict data from the OGM. Blue and yellow lines represent DLE-1 labeled areas on the patient OGM map, or the reference chromosomes, grey lines indicate the patient map as it aligns to the reference genome. The orange triangle depicts the region from the OGM map that was deleted compared to the reference. The bottom three tracks show the corresponding locations in WGS data and the 50% decrease in read coverage at IKZF1 indicating a heterozygous deletion.

Several of the previous studies have noted that OGM alone identifies the vast majority of rearrangements identified by classic cytogenetics and, in some cases, reveals SVs that are missed by karyotyping [30]. In this study, we compared the SVs identified by WGS and OGM to those reported by cytogenetics in the nine cases in which karyotype data were available (Table 1). With the exceptions noted below, WGS, OGM or both confirmed the SVs reported by cytogenetics, including BCR::ABL1 translocations and IKZF1 and CDKN2A deletions. In one case, W31, cytogenetics reported wild-type (WT) IKZF1, while both WGS and OGM reported a partial deletion and WGS confirmed the deletion as heterozygous (Figure 5).

**Figure 6:**
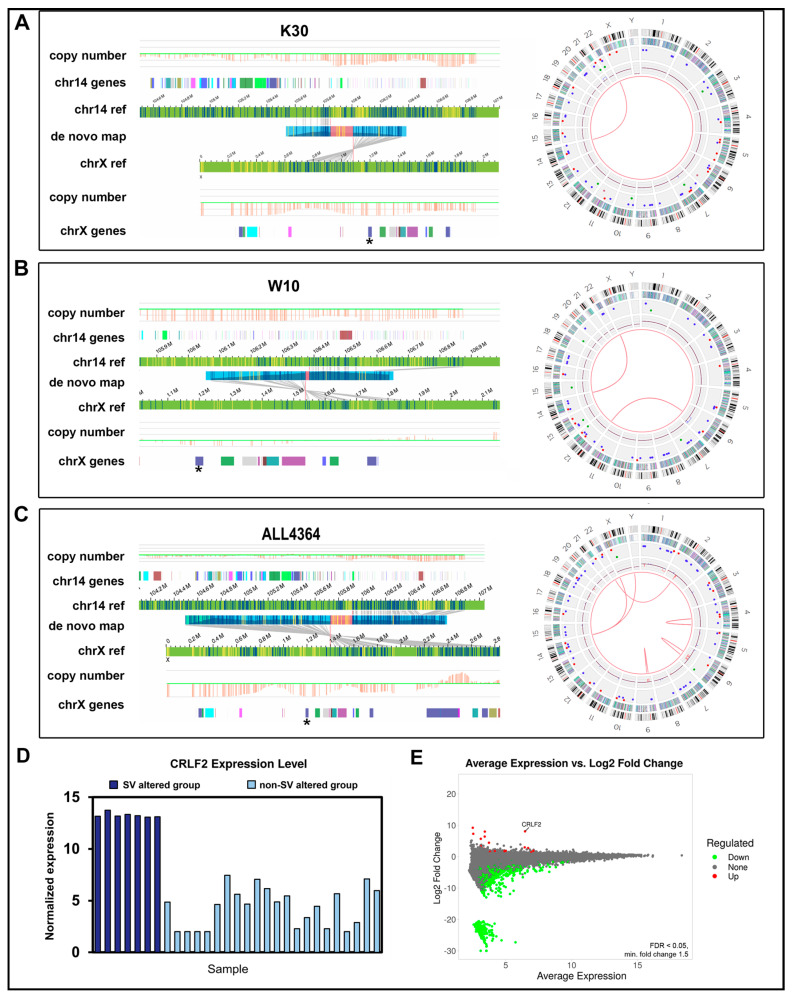
OGM-derived circos plots and t(14;X) translocations with breakpoints near *CRLF2* and IGH regions. (**A**–**C**) Shown are translocation visualization and circos plots from OGM analysis of three samples with *IKZF1* deletions who also have t(14;X) translocations near *CRLF2*. Right: Track labels for circos plots are from outside to inside: chromosome number, cytoband, gene regions, SV (blue = deletion, red = insertion, pink = inversion, green = duplication), copy number, translocations. Left: OGM maps identifying translocation. Tracks are specified on the left. The *CRFL2* gene region is marked with an asterisk on chrX. Blue and yellow lines represent DLE-1 labeled areas on the patient OGM map or the reference chromosomes; grey lines indicate the alignment of the patient map to the reference genome. The pink area on the de novo patient map represents an unalignable junction. (**D**) Normalized RNA-Seq expression values for 7 individuals containing CRLF2 rearrangements or nearby SVs (black box) versus those with wild-type CRLF2. (**E**) CRLF2 is upregulated in individuals with CRLF2 rearrangements or nearby SVs. MA plot showing average expression and log2 fold change FDR <0.05 and a minimum fold-change of 1.5.

The *IGH::CRLF2* translocation reported by cytogenetics in sample ALL4364 was detected by OGM using the de novo pipeline, but in contrast to cytogenetics, OGM determined that the breakpoint occurred ≈200 kb away from the *CRLF2* gene. The WGS data did not report an *IGH::CRLF2* translocation due to an insufficient number of reads passing filtering criteria. We observed *IGH::CRLF2* translocations in two additional Hispanic individuals, W10 and K30, by OGM using the de novo pipeline (Figure 6). To our knowledge, for two of the three individuals, W10 and K30, cytogenetics did not report an *IGH::CRLF2* translocation. RNA-Seq did not show any expression of an *IGH::CRLF2* gene fusion event in samples ALL4364, W10, or K30. However, RNA-Seq data indicated that all three samples had increased CRLF2 transcription, suggesting that the translocations placed CRLF2 expression under control of the strong IGH enhancer (Figure 6D,E). Thus, while OGM plus WGS identified most of the structural rearrangements reported by cytogenetics and identified several clinically relevant SVs missed by cytogenetics, several SVs identified by cytogenetics could not be confirmed by OGM or WGS.

Repetitive regions pose particular difficulties for SV identification for OGM and WGS and are likely the primary reason these methods fail to confirm certain SVs identified by cytogenetics. The *CRLF2* gene resides in the pseudoautosomal regions (PAR) of chrX/Y, rendering it difficult for OGM molecules and especially short-reads from WGS to be aligned to the appropriate region on chrX or chrY. For example, OGM ambiguously mapped an *IGH::CRLF2* translocation to both t(14;X) and t(14;Y) due to *CRLF2* location in the PAR region of chrX and chrY (Figure 6C). Likewise, the *IGH* region undergoes somatic rearrangements during B-cell development, such as V(D)J recombination [31]. These rearrangements generate a diverse repertoire of antibody genes and homologous regions that result in mapping ambiguities such as distinguishing between different *IGH* gene segments and can complicate the identification and characterization of SVs in B-cells, especially WGS.

### 3.5. Integrating WGS, OGM, and RNA-Seq for Gene Fusion Detection

**Figure 7:**
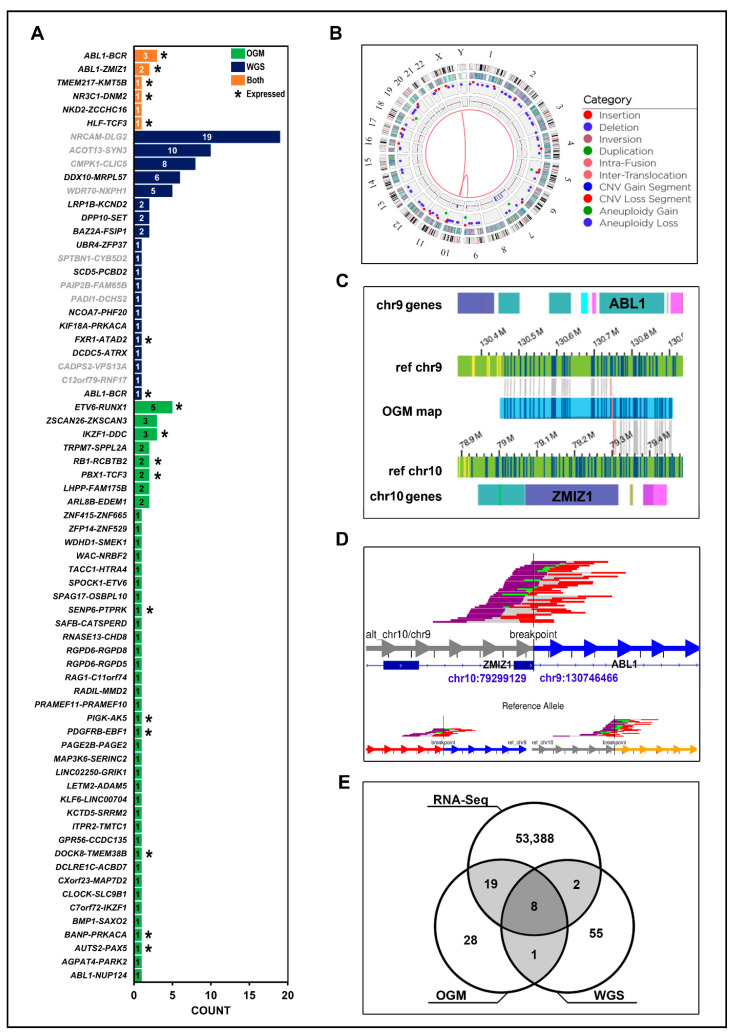
A more comprehensive gene-fusion landscape in B-ALL: (**A**) Putative gene fusion events identified by either WGS, OGM, or both technologies. Waterfall plot depicts counts of annotated gene fusion events using OGM’s in-house annotations (green), WGS Oncofuse annotated breakpoints (blue), or both technologies (orange). WGS fusion pairs with a breakpoint in a repeat region are greyed out. Fusion pairs with evidence of expression in ≥1 sample (≥16 supporting RNA-Seq reads) are marked with an asterisk. (**B**) OGM circos plot from sample PAWFUU using the RVP showing a ABL1::ZMIZ1 t(9;10) translocation. Track labels are as follows from outside to inside: chromosome number, cytoband, gene regions, SV (blue = deletion, red = insertion, pink = inversion, green = duplication), copy number, translocations. (**C**) View of the *ABL1::ZMIZ1* fusion in OGM data in the same individual. Blue and yellow lines represent DLE-1 labeled areas on the patient OGM map or the reference chromosomes; grey lines indicate the patient map as it aligns to the reference genome. (**D**) WGS split-read alignments indicating reads that map to two different regions of the genome, between chr9 and chr10, supporting an *ABL1::ZMIZ1* fusion at intron 1 of *ABL1* and exon 16 of *ZMIZ1* in the same individual. The reads supporting the reference allele are depicted on the bottom panel. Reads aligning to the minus strand are red and those aligning to the plus strand are purple. The un-sequenced space between read pairs is depicted by gray bars. Green reads mean overlap of mate pair reads. (**E**) Gene fusion events detected by WGS, OGM, and RNA-Seq in 29 B-ALL individuals and the overlap of those fusion events detected using two methods. The 8 fusions with expression data are listed in Table 2.

Both OGM and WGS identified a number of gene fusion events in our cohort, although only a few in common (Figure 7 and Table 2). Overall, 56 fusions were reported by OGM, 66 by WGS, and 9 by both technologies. RNA-Seq data provided confirmation of the fusion event and its expression in 48% of OGM predicted fusions and 15% of those predicted by WGS. We identified 8 gene fusion events in our cohort that were able to be validated by all three techniques and 9 fusions confirmed by OGM and WGS (Figure 7). The fusions only detected by one technology highlight the importance that using both WGS and OGM can have on obtaining a comprehensive SV landscape in B-ALL as many fusions go undetected by using either technique in isolation.

With regard to those fusions identified by all three methods (Table 2), we note that one rare fusion between *ABL1::ZMIZ1*, t(9;10) was reported in two individuals, PAWFUU (Figure 7) and PAVCYL (Supplementary Table S3). Although *ABL1* is known to have more than 76 fusion partners, including *ZMIZ1*, the fusion is rare and only two other case reports of this translocation have been described since 2008 [32,33,34]. Previously, it was shown that exon 14 of *ZMIZ1* was fused to exon 2 of *ABL1* containing one of the proline-rich domains of *ZMIZ1* and the tyrosine kinase domain of ABL1 [32,33,34]. It is likely that this fusion transcript yields the synthesis of a fusion protein similar to the oncogenic tyrosine kinase *BCR::ABL1*, since both reported cases saw a favorable outcome with treatment of tyrosine kinase inhibitor, dasatinib. Figure 7D shows WGS reads for the fusion of intron 1 of *ABL1* and exon 16 of *ZMIZ1* in one Hispanic individual in our cohort, PAWFUU. A corresponding OGM map (Figure 7C) confirms the fusion.

### 3.6. Detection of Novel Gene Fusion Events with OGM

**Figure 8:**
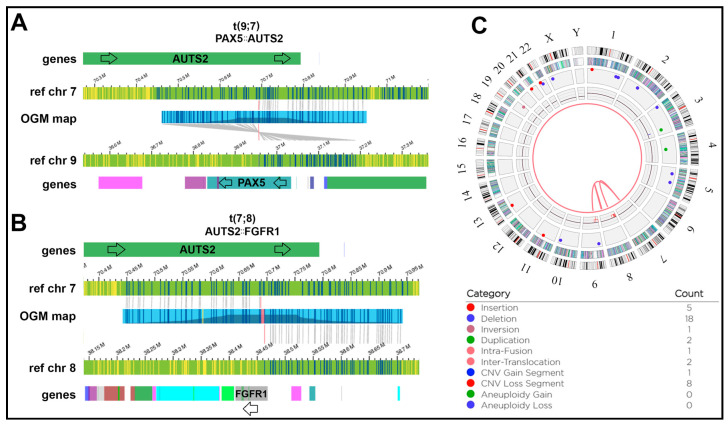
OGM complex rearrangement not reported by DELLY in a Hispanic individual: W13: (**A**) t(7;9) inverted translocation causing a *PAX5::AUTS2* fusion event. Blue and yellow lines represent DLE-1 labeled areas on the patient OGM map or the reference chromosomes; grey lines indicate the patient map as it aligns to the reference genome. (**B**) The same individual carries a t(7;8) translocation causing juxtaposing *FGFR1* and *AUTS2* but in opposite orientations and ultimately not leading to a gene fusion. Both rearrangements were detected with the OGM rare variant pipeline (RVP). The pink area on the OGM map indicates a small unalignable junction between the breakpoints. (**C**) Circos plot view of the t(7;8) and t(7;9) translocations. Track labels are as follows from outside to inside: chromosome number, cytoband, gene regions, SV (blue = deletion, red = insertion, pink = inversion, green = duplication), copy number, translocations.

A number of fusions were identified by OGM and confirmed by RNA-Seq but not by WGS, several of which were novel. We deemed a putative fusion as novel if it was not reported in ChimerDB 4.0, FusionGDB 2.0 databases or the manual literature curation [32,35]. FusionGDB 2.0 includes gene fusion information from Entrez mRNA sequence libraries and TCGA data from ChiTars 5.0. human tumors [36]. Two translocations t(7;9) and t(7;8) involving *AUTS2* occurred in one Hispanic individual. One generated a *PAX5::AUTS2* gene fusion and the other linked *FGFR1* to *AUTS2* but in an inverted orientation of the genes (Figure 8). The RNA-Seq data showed only low-level expression of the *PAX5::AUTS2* fusion, with only five supporting read pairs. The *PAX5::AUTS2* fusion in WGS was only reported by LUMPY and not DELLY, and the *AUTS2-FGFR1* putative fusion did not have any supporting split reads and was also only detected by LUMPY. The low expression level of *PAX5::AUTS2* may suggest that that allele fraction of the fusion is too low to be detectable at 40X WGS. Although the *PAX5::AUTS2* translocation is recurrent in pediatric B-ALL, *AUTS2* was not previously reported to fuse with *FGFR1* [37,38]. *FGFR1*, a fibroblast growth factor receptor, has been reported to fuse with *BCR* in other B-ALL cases, causing expression of a dominant fusion protein resistant to tyrosine kinase inhibitors, but its fusion with *AUTS2* remains uncharacterized [39,40]. Since the two genes are in inverted orientation in the fusion, this rearrangement may cause the inactivation of both genes, rather than hyperactivation of *FGFR1*.

Other novel putative gene fusion events are listed in Supplementary Table S3. These results demonstrate that OGM data alone can yield valuable information on gene fusion events, including the identification of novel fusions. When supplemented with RNA-Seq data, putative gene fusions from OGM data that show expression can be targeted as prognostic indicators or as future therapeutic targets.

## 4. Discussion

In this study, we combined OGM, WGS, and RNA-Seq data for the detection of somatic SVs in 29 pediatric B-ALL individuals. While WGS is considered the gold standard, we proposed a computational method for integrating OGM and RNA-Seq data to better characterize the landscape of SVs. OGM is proficient at resolving complex SVs, especially in repetitive regions of the genome but is limited in reporting the precise breakpoints of SVs. When an SV occurs, the location of the nearest DLE-1 label is reported in lieu of a precise breakpoint. WGS is able to refine SV breakpoints down to single base pair precision but suffers from mapping errors over large insertions or repetitive elements and other low mappability regions. Unlike OGM, in which SVs may be physically observed on long-molecules of DNA, SV callers designed for short-read data must predict/infer the location and type of SVs based on the orientation and distance of paired-end reads and, thus, may yield more false-positives when short-reads map to multiple genomic positions with uncertainty. RNA-Seq is able to provide gene expression data and further determines if predicted gene fusion events show any expression, making those fusions potential therapeutic targets.

We showed that the integration of OGM, WGS, and RNA-Seq uncovers SVs not previously seen when using either technology in isolation. Those SVs that are detected in both WGS and OGM represent only a small percentage (11.6%) of the total somatic SVs occurring in 29 pediatric B-ALL individuals. Our results are in line with two previous reports comparing short-read WGS to OGM data in single individuals [16,17]. Among this small overlap, 20.7% of deletions, 11.8% of duplication/gains, and 2.2% of translocations are detected by both technologies. Some of this difference is due to the size sensitivities of the techniques. OGM cannot confidently resolve SVs <500 bp, while the majority of deletions detected by WGS are <500 bp. The same comparison cannot be applied to insertions since insertion sizes are not reported by WGS callers LUMPY and DELLY owing to short paired-end reads often being unable to span both ends of large insertions. OGM is able to resolve >500 insertions, ranging from 10–500 kb. Translocations are readily detected by WGS, OGM, and RNA-Seq, many causing relevant gene-fusion events. WGS detects many more translocations than OGM but also reports ambiguous breakpoints that resolve as nearby insertion events in OGM data. We found that 18.9% of all translocations identified by WGS in our cohort were adjacent to an OGM insertion breakpoint located ≤100 kb away. In many cases, these breakpoints overlap repeat elements defined by UCSC, indicating the challenges repeat regions pose in short-read NGS data.

We further investigated translocations and other SVs that cause gene-fusion events with the aim of discovering novel fusion events and any evidence of expression. We showed that several gene fusion events predicted by WGS and OGM have not previously been reported and some showed expression in RNA-Seq data, marking them as potential clinical targets. Several more novel fusions, though not expressed, remain of interest due to the ability of gene fusions to abrogate both genes involved in the event leading to the potential downregulation of tumor suppressors or regulatory genes.

Disagreement between OGM and WGS around gene fusions may occur due to the way Bionano reports coordinates for corresponding SVs, including gene fusions. In lieu of the exact chromosomal position, the location of the nearest DLE-1 site is reported. As a result, gene fusion events from OGM may be reported within a few kilobases of the actual location. The quality of DLE-1 labeling during library preparation may additionally influence how many DLE-1 labels are available for reporting on the patient map. In this scenario, Bionano reports the nearest gene without annotating the SV call as a gene fusion [18]. WGS is able to resolve a fraction of these with base pair precision, but DELLY and LUMPY each suffer from a higher number of false-positive translocation calls that correlate with increasing coverage depth, as compared to OGM, especially in repetitive regions. Inferring gene-fusion events from short reads in RNA-Seq data is also challenging, although recent efforts are attempting to refine the method [41]. Unlike genome approaches, RNA-Seq focuses only on expressed regions. In order to maintain a low incidence of false-positive results, the implementation of stringent filters becomes important. However, this approach inadvertently leads to the exclusion of genuine driver fusions if they exhibit lower expression levels or are present at a low allele frequency. This is particularly relevant in cases involving heterogeneous tumor samples or samples with inadequate RNA-Seq coverage. Furthermore, gene-fusion events that are not expressed are not available in RNA-Seq data, making the use of WGS and OGM particularly informative in this situation. We obtained a total of ≈53.3 million RNA-Seq reads per sample replicate (n = 3) and retained fusion events in the analysis if they had ≥16 supporting discordant reads, resulting in >53,000 fusions across 29 B-ALL samples (Figure 7E). In total, 88.8% (8/9) of gene fusions identified by OGM and WGS also showed expression in RNA-Seq (Figure 7E and Table 2), potentially indicating these as candidates in functional applications. Unsurprisingly, we observed expression of *BCR::ABL1* translocations as this fusion is characteristic of B-ALL and a well-known oncogenic fusion protein that is presently treated with imatinib, a tyrosine kinase inhibitor [42]. These results suggest that the workflow of WGS, OGM, and RNA-Seq could be used to identify established and novel fusion genes that are expressed and, thus, could be clinically targeted.

This study provides a comprehensive workflow for integrating WGS, Bionano OGM, and RNA-Seq data for the discovery of SVs and demonstrates how SV detection can be expanded using these methodologies. Future studies should aim to incorporate long-read sequencing technologies seamlessly with OGM data. While long-read sequencing suffers in sequencing accuracy compared to short-read data, it could more accurately be compared to OGM data while still providing the sensitivity for smaller SVs that are beyond the limits of detection in OGM. In addition, there presently exists no standard workflow for the integration of WGS and OGM data. Since sequencing is still the gold standard in clinic, the development of computational pipelines that aim to integrate OGM data with current clinical gold standards will be a necessity for improving clinical diagnostics in future research, as better resolution of SVs can help in developing personalized medicine by uncovering new therapeutic targets.

## Figures and Tables

**Table 1:** A comparison of known cytogenetic features to WGS and OGM calls in pediatric B-ALL samples.

Sample	Known Cytogenetics	WGS	OGM	Differences from Cytogenetics
W0	IKZF1 deletion	IKZF1 deletion confirmed.	IKZF1 deletion confirmed.	Concur
W10	IKZF1 deletion	IKZF1 deletion confirmed.	IKZF1 deletion confirmed. IGH::CRLF2 translocation	OGM reports IGH::CRLF2 translocation
W13	IKZF1 wild type	Normal IKZF1 confirmed	Normal IKZF1 confirmed	Concur
W31	IKZF1 wild type	IKZF1 deletion reported	IKZF1 deletion reported	WGS and OGM report deletion in IKZF1 (Figure 5)
MXP3	BCR::ABL1 translocation, IKZF1 deletion, PAX5 deletion	IKZF1 and PAX5 deletions confirmed. BCR::ABL1 translocation confirmed	IKZF1 and PAX5 deletions confirmed. BCR::ABL1 translocation confirmed	Concur
ICN1	BCR::ABL1 translocation	BCR::ABL1 translocation confirmed	BCR::ABL1 translocation confirmed	Concur
ALL4364	IGH::CRLF2	IGH::CRLF2 translocation reported but does not pass filtering	abParts::KIAA0125 translocation near but not involving CRLF2	OGM and WGS do not report IGH::CRLF2 but OGM identifies a nearby translocation
PVCRK	IGH::EPOR translocation, CDKN2A, IKZF1, and JAK2 deletions	No IGH::EPOR translocation reported. CDKN2A and IKZF1 deletion confirmed. No JAK2 SV reported.	No IGH::EPOR translocation. IKZF1, PAX5, and CDKN2A deletions confirmed. No JAK2 SV reported	Additional PAX5 deletion identified by OGM. No IGH::EPOR translocations reported in WGS or OGM. No JAK2 SV reported by OGM or WGS
PAVDRS	IGH::EPOR, CDKN2A, IKZF1, and PAX5 deletions	No IGH::EPOR translocation. IKZF1, CDKN2A deletions confirmed. No PAX5 deletion reported	No IGH::EPOR translocation. IKZF1, CDKN2A, and PAX5 deletions confirmed.	WGS does not report the PAX5 deletion

**Table 2:** Summary of gene fusions detected by both WGS and OGM and any evidence of corresponding expression from RNA-Seq.

Fusion	Sample	Location	OGM	WGS	RNA-Seq
*TMEM217::KMT5B*	ALL-19	chr6:37,243,578 > chr11:68,174,277	✓	✓	✓
*BCR::ABL1*	G2650	chr9:130,854,197 > chr22:23,219,813	✓	✓	✓
*NR3C1::DNM2*	G2650	chr5:143,350,483 > chr19:10,739,595	✓	✓	✓
*BCR::ABL1*	ICN1	chr9:130,846,798 > chr22:23,290,277	✓	✓	✓
*BCR::ABL1*	MXP3	chr9:130,821,448 > chr22:23,290,361	✓	✓	✓
*ABL1::ZMIZ1*	PAVCYL	chr9:130,746,469 > chr10:79,299,033	✓	✓	✓
*HLF::TCF3*	ALL-07	chr17:55,319,005 > chr19:1,619,186	✓	✓	✓
*ABL1::ZMIZ1*	PAWFUU	chr9:130,746,466 > chr10:79,299,129	✓	✓	✓
*NKD2::ZCCHC16*	ICN1	chr5_KI270792v1alt:30755 > chrX:112,294,171	✓	✓	✕

## Data Availability

WGS, RNA-Seq, and Bionano data can be accessed at: https://doi.org/10.26208/beyz-cz18, accessed on 6 March 2024. or at the European Genome Phenome archive, or by contacting the corresponding author and The Penn State Institute of Personalized Medicine: https://research.med.psu.edu/departments/personalized-medicine/, accessed on 6 March 2024.

## Supplementary Materials

The following supporting information can be downloaded at: https://www.mdpi.com/article/10.3390/jpm14030291/s1, Figure S1: Comparison of SVs in patient primary tumor and expanded PDX model from Bionano de novo analysis; Figure S2: Human CD45 cell engraftment in animal Bone marrow (BM) and Spleens (SP). Mice were terminated when sick or moribund post-injection of human cells or PDXs and their BM and SP were harvested. After tissue cell dissociation and processing, single cells were stained for mCD45 and hCD45; Table S1: B-ALL cohort details; Table S2: Comparison of WGS, OGM, karyotyping, FISH, and RNA-Seq for SV detection; Table S3: Novel putative gene-fusion events identified by WGS, OGM, or both.
