# Supplementary material for: Integrating Optical Genome Mapping and Whole Genome Sequencing in Somatic Structural Variant Detection
Source: J Pers Med. 2024 Mar 9;14(3):291. doi: 10.3390/jpm14030291 (PMC10971281; doi:10.3390/jpm14030291)
Supplement: Supplementary file 1 [file jpm-14-00291-s001.zip › jpm-2887902-supplementary.pdf]

*Supplementary Materials*

**Supplementary Table S1.** Clinically available data of B-ALL cohort. Principal component analysis of ancestry results: AMR = admixed American, EAS = east Asian, SAS = south Asian, EUR = European.

| Individual | Leukemia Type | Self-reported race (if available) | PCA Ancestry Classification | Age (yrs) | Gender | Original cytogenetic features reported |
|---|---|---|---|---|---|---|
| W0 | B-ALL | Hisp/Mex | AMR | 10 | M | CRLF2+ low. IKZF1del |
| W10 | B-ALL | Hisp/Mex | AMR | 18 | M | CRLF2+ high, IKZF1 del |
| W31 | B-ALL | Hisp/Mex | AMR | 10 | M | CRLF2+ IKZF1 wt |
| W13 | B-ALL | Hisp/Mex | AMR | 18 | M | CRLF2- IKZF1 wt |
| G02 | B-ALL | Hisp/Mex | AMR | 9 | F | CRLF2+ CD34+ |
| E13 | B-ALL | Hisp/Mex | AMR | 6 | F | CRLF2+ CD34+ |
| K30 | B-ALL | Hisp/Mex | AMR | 12 | F | CRLF2+ CD34+&CD34- |
| K09 | B-ALL | Hisp/Mex | AMR | | F | |
| PAVDRS | B-ALL | | AMR | 9 | M | IGH-EPOR, CDKN2A, IKZF1, PAX5 |
| ALL4364 | B-ALL | | AMR | 4 | M | JAK2 R683G, IGH-CRLF2 |
| PAWFUU | B-ALL | | AMR | 18 | F | RANBP2-ABL1 |
| PAVCYL | B-ALL | | AMR | 8 | M | ZMIZ1-ABL1 |
| G2650 | B-ALL | | AMR | >20 | M | |
| LAX7 | B-ALL | | AMR | >20 | M | IKZF1 del CD10+CD19+ |
| ICN1 | B-ALL | | EAS | 14 | F | BCR-ABL1 |
| ALL-17 | B-ALL | | EAS | 8 | F | |
| PAVVIE | B-ALL | | EUR | 5 | M | NUP153-ABL1 |
| PVCRK | B-ALL | | EUR | >20 | M | IGH-EPOR, CDKN2A, IKZF1, JAK2 |
| ALL-07 | B-ALL | | EUR | 7 | M | |
| ALL-11 | B-ALL | | EUR | 3 | F | |
| ALL-19 | B-ALL | | EUR | 16 | M | |
| ALL-25 | B-ALL | | EUR | 12 | M | |
| ALL-53 | B-ALL | | EUR | 7 | F | |
| ALL-57 | B-ALL | | EUR | 5 | F | |
| ALL-59 | B-ALL | | EUR | 13 | F | |
| ALL-82 | B-ALL | | EUR | 5 | F | |
| ALL-02 | B-ALL | | EUR | 5 | F | |
| ALL-58 | B-ALL | | EUR | 2 | F | |
| MXP3 | B-ALL | | SAS | 13 | M | |

**Supplementary Table S2.** Comparison of WGS, OGM, Karyotyping, FISH, and RNA-Seq techniques for detection of SVs.

| Variant type | WGS | OGM | Karyotype | FISH | RNA-Seq |
|---|---|---|---|---|---|
| SNV | ✓ | N/A | N/A | N/A | ✓ |
| Deletion | ✓ | >500 bp | >5-10 Mb | targeted | Expression-dependent |
| Duplication | ✓ | >500 bp | >5-10 Mb | targeted | Expression-dependent |
| Inversion | ✓ | >500 bp | >5-10 Mb | targeted | Expression-dependent |
| Insertion | small | >500 bp | >5-10 Mb | targeted | Expression-dependent |
| Translocation | ✓ | >500 bp | >5-10 Mb | targeted | Expression-dependent |
| Aneuploidy | ✓ | ✓ | ✓ | targeted | Expression-dependent |
| Repeat expansion | N/A | ✓ | N/A | N/A | N/A |
| Loss of heterozygosity | ✓ | ✓ | N/A | N/A | Expression-dependent |

**Supplementary Table S3**. Novel putative gene-fusion events identified by WGS, OGM, or both. Novel fusions were not reported in ChimerDB 4.0, FusionGDB 2.0 databases or manual literature curation.

| Fusion genes | Putative novel: not in FusionGDB, ChimerDB, or lit search | Previously reported | RNA-Seq expression (≥16 supporting reads) | Fusion genes (contd.) | Putative novel (contd.) | Previously reported (contd.) | RNA-Seq expression (contd.) |
|---|---|---|---|---|---|---|---|
| ABL1::NUP214 | | ✓ | ✓ | NCOA7::PHF20 | ✓ | | |
| ABL1::ZMIZ1 | | ✓ | ✓ | NKD2::ZCCHC16 | ✓ | | |
| ACOT13::SYN3 | | ✓ | | NR3C1::DNM2 | ✓ | | ✓ |
| AGPAT4::PARK2 | | ✓ | | PADI1::DCHS2 | ✓ | | |
| ARL8B::EDEM1 | ✓ | | | PAGE2B::PAGE2 | ✓ | | |
| AUTS2::PAX5 | | ✓ | | PAIP2B::FAM65B | ✓ | | |
| BANP::PRKACA | ✓ | | ✓ | PBX1::TCF3 | | ✓ | |
| BAZ2A::FSIP1 | | ✓ | | PDGFRB::EBF1 | | ✓ | |
| BMP1::SAXO2 | ✓ | | | PIGK::AK5 | ✓ | | ✓ |
| C12orf79::RNF17 | ✓ | | | PRAMEF11::PRAMEF10 | ✓ | | |
| C7orf72::IKZF1 | ✓ | | | RADIL::MMD2 | | ✓ | |
| CADPS2::VPS13A | ✓ | | | RAG1::C11orf74 | ✓ | | |
| CLOCK::SLC9B1 | ✓ | | | RB1::RCBTB2 | | ✓ | |
| CMPK1::CLIC5 | | ✓ | | RGPD6::RGPD5 | | ✓ | ✓ |
| CXorf23::MAP7D2 | ✓ | | | RGPD6::RGPD8 | | ✓ | |
| DCDC5::ATRX | | ✓ | | RNASE13::CHD8 | ✓ | | |
| DCLRE1C::ACBD7 | ✓ | | | SAFB::CATSPERD | ✓ | | |
| DDX10::MRPL57 | | ✓ | | SCD5::PCBD2 | ✓ | | |
| DOCK8::TMEM38B | ✓ | | ✓ | SENP6::PTPRK | ✓ | | ✓ |
| DPP10::SET | ✓ | | | SPAG17::OSBPL10 | ✓ | | |
| ETV6::RUNX1 | | ✓ | ✓ | SPOCK1::ETV6 | ✓ | | |
| FXR1::ATAD2 | ✓ | | ✓ | SPTBN1::CYB5D2 | ✓ | | |
| GPR56::CCDC135 | | ✓ | | TACC1::HTRA4 | ✓ | | |
| HLF::TCF3 | | ✓ | ✓ | TMEM217::KMT5B | ✓ | | ✓ |
| IKZF1::DDC | | ✓ | ✓ | TRPM7::SPPL2A | | ✓ | |
| ITPR2::TMTC1 | | ✓ | | UBR4::ZFP37 | | ✓ | |
| KCTD5::SRRM2 | | ✓ | | WAC::NRBF2 | ✓ | | |
| KIF18A::PRKACA | ✓ | | | WDHD1::SMEK1 | ✓ | | |
| KLF6::LINC00704 | ✓ | | | WDR70::NXPH1 | ✓ | | |
| LETM2::ADAM5 | | ✓ | | ZFP14::ZNF529 | ✓ | | |
| LHPP::FAM175B | ✓ | | | ZNF415::ZNF665 | ✓ | | |
| LINC02250::GRIK1 | ✓ | | | ZSCAN26::ZKSCAN3 | ✓ | | |
| LRP1B::KCND2 | ✓ | | | | | | |
| MAP3K6::SERINC2 | ✓ | | | | | | |

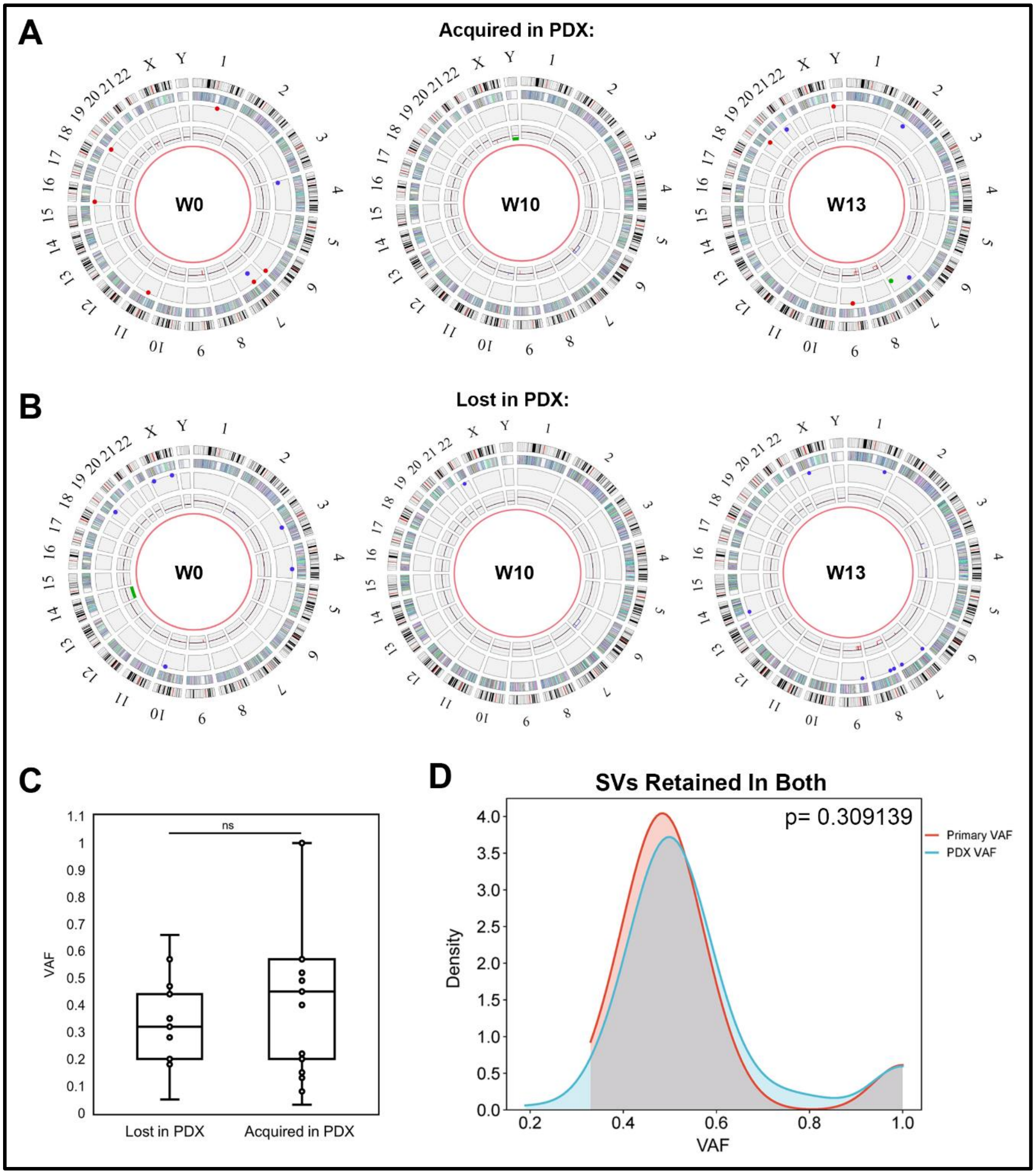


**Supplementary Figure S1** Comparison of SVs in patient primary tumor and expanded PDX model from Bionano de novo analysis:

**(A)** Circos plots of SVs acquired in PDX model. SVs were originally not present/detectable in primary tumor but present/detectable in PDX. Track labels are as follows from outside to inside: chromosome number, cytoband, gene regions, SV (blue=deletion, red=insertion, pink=inversion, green=duplication), copy number, translocations. **(B)** Circos plots of SVs lost in PDX model, originally present/detectable in primary tumor, but not in PDX model. **(C)** Variant allele frequencies of SVs either lost or acquired in the PDX model compared to the primary tumor. Middle line represents median (p=0.13456, two-tailed T-test.) D) Density plot representing variant allele frequencies of SVs retained in both patient primary tumor and PDX model (p=0.309139, two-tailed T-test).

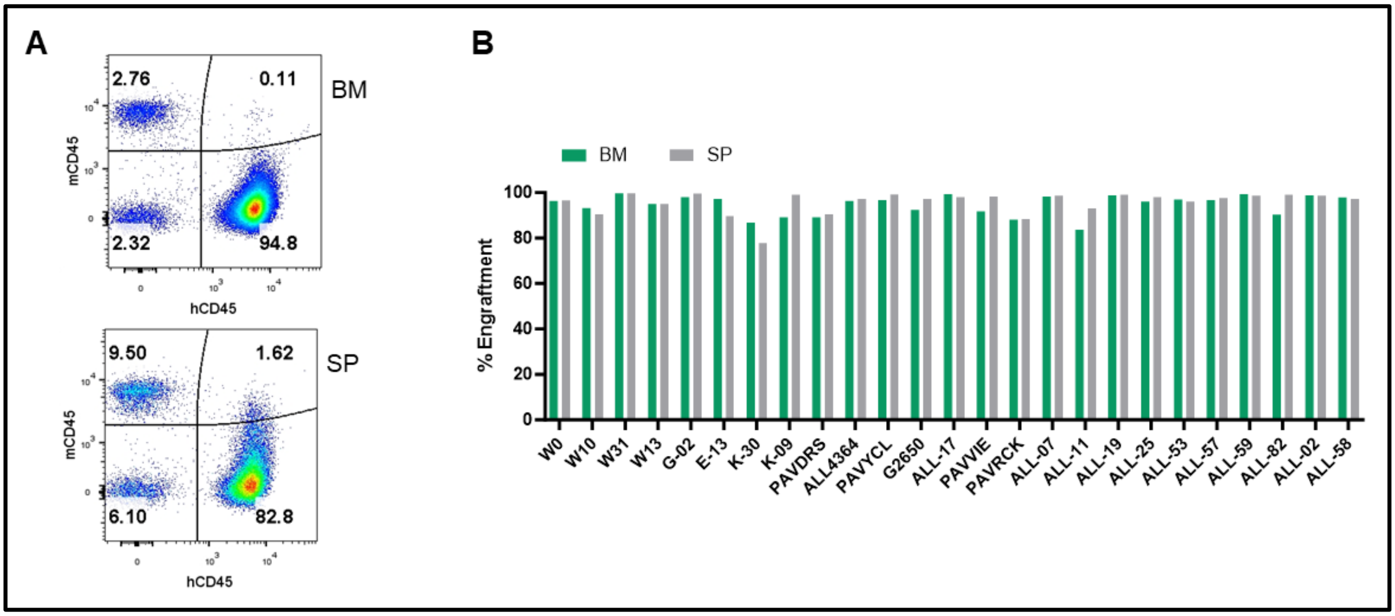


**Supplementary Figure S2** Human CD45 cell engraftment in animal Bone marrow (BM) and Spleens (SP). Mice were terminated when sick or moribund post-injection of human cells or PDXs and their BM and SP were harvested. After tissue cell dissociation and processing, single cells were stained for mCD45 and hCD45. **(A)** A representative flow dot plot of BM (top) and SP(bottom) from PDX E-13 is shown. (**B)** The human CD45 cell engraftment data based on staining of mCD45 and hCD45 are presented in the bar graph. Human cell engraftment is defined as the percentage of human CD45+ cells divided by the percentage of total mouse and human CD45+ cells multiplied by 100.
